# Ero1–PDI interactions, the response to redox flux and the implications for disulfide bond formation in the mammalian endoplasmic reticulum

**DOI:** 10.1098/rstb.2011.0403

**Published:** 2013-05-05

**Authors:** Adam M. Benham, Marcel van Lith, Roberto Sitia, Ineke Braakman

**Affiliations:** 1 School of Biological and Biomedical Sciences, Durham University, South Road, Durham DH1 3LE, UK; 2 College of Medical, Veterinary and Life Sciences, Davidson Building, University of Glasgow, Glasgow G12 8QQ, UK; 3 Università Vita-Salute San Raffaele, DiBiT, Via Olgettina 58, 20132 Milano, Italy; 4 Division of Genetics and Cell Biology, San Raffaele Scientific Institute (OSR), Milan, Italy; 5 Cellular Protein Chemistry, Faculty of Science, Utrecht University, Padualaan 8, 3584 Utrecht, The Netherlands

**Keywords:** chaperone, endoplasmic reticulum, protein folding, redox, disulfide bond

## Abstract

The protein folding machinery of the endoplasmic reticulum (ER) ensures that proteins entering the eukaryotic secretory pathway acquire appropriate post-translational modifications and reach a stably folded state. An important component of this protein folding process is the supply of disulfide bonds. These are introduced into client proteins by ER resident oxidoreductases, including ER oxidoreductin 1 (Ero1). Ero1 is usually considered to function in a linear pathway, by ‘donating’ a disulfide bond to protein disulfide isomerase (PDI) and receiving electrons that are passed on to the terminal electron acceptor molecular oxygen. PDI engages with a range of clients as the direct catalyst of disulfide bond formation, isomerization or reduction. In this paper, we will consider the interactions of Ero1 with PDI family proteins and chaperones, highlighting the effect that redox flux has on Ero1 partnerships. In addition, we will discuss whether higher order protein complexes play a role in Ero1 function.

## 1. Oxidative protein folding in the endoplasmic reticulum

Disulfide bonds are formed between two cysteine residues, either within proteins (intramolecular disulfides) or between proteins (intermolecular disulfides) [1]. This is a rare occurrence in the cell cytosol because the reducing environment favours free thiols (–SH). However, in the bacterial periplasm [2], the mitochondrial intermembrane space and the eukaryotic endoplasmic reticulum (ER [3]), the environment is more oxidizing and favours disulfide bond formation (S–S). In the ER, native disulfide bonds are integrated into proteins early during the folding process [4]. This occurs while the protein is being threaded through the translocon during, or shortly after, translation. The process of ensuring that a protein is properly folded and equipped with the correct disulfide bond arrangements is carefully coordinated by various protein disulfide isomerases (PDIs), oxidoreductases, chaperones and other folding factors [5]. These folding assistants ensure that disulfide bond formation is coupled to other post-translational modifications, such as the introduction of N-linked glycans and to quality control processes [6]. The main players involved in oxidative folding and quality control have been identified, but how they interact and work together in a coordinated fashion is not fully understood. The ER can be viewed as the control point for a cell's secretory output and for the integrity of proteins within the secretory pathway itself. Thus, understanding how the ER machinery works in different cells and tissues is essential for us to tackle various biological problems, ranging from diseases of misfolding to the need for improved production of recombinant proteins.

## 2. Protein disulfide isomerase and Ero1

Two of the major contributors to disulfide bond formation in the ER are PDI and the ER oxidoreductin (or oxidoreductase) Ero1. There is a solitary Ero1 protein in *Saccharomyces* *cerevisiae* (Ero1p [7,8]) and two (Ero1α and Ero1β [9,10]) in mammals. Together, PDI and Ero1 proteins harness the oxidizing power of molecular oxygen to create de novo disulfide bonds in a newly folding protein [11,12]. The exchange of disulfide bonds from Ero1 to PDI to client necessitates electron flow in the reverse direction, from client to PDI to Ero1. Ero1s use the cofactor flavin adenine dinucleotide (FAD) to reduce molecular oxygen, generating peroxide in the process [13,14]. PDI is able to supply, rearrange (isomerize) or reduce disulfide bonds in a client protein [15]. The ability of PDI to perform these functions depends on its two redox-active **a** and **a**′ thioredoxin domains [16]. The **a** type domains are separated by two redox-inactive **b** domains in an **abb**′**xa**′ arrangement [17,18], where the **x** linker region contributes to mobility and modulates client access to PDI [19,20]. The PDI **a** type domains have CGHC active sites: their high biochemical reduction potential (−180 mV) makes PDI thermodynamically suited for donating disulfide bonds to reduced protein clients [21]. During disulfide bond formation in mammalian cells, the **a** domain of PDI is oxidized by its **a**′ domain [22] after the **a**′ domain of PDI has been preferentially oxidized by Ero1α [23,24]. The C_94_xxxxC_99_ region (x = any amino acid), on a flexible loop of Ero1α, effects the transfer of disulfide bonds from Ero1α to the **a**′ domain of PDI [25]. In turn, the C_94_xxxxC_99_ site of Ero1α receives a disulfide bond from the C_394_xxC_397_ site, which is in direct communication with the FAD moiety [26]. A similar mechanism occurs in *S. cerevisiae* [27]; however, in yeast, Pdi1p is glycosylated, and in the context of the full-length protein, the **a** domain functions better as an isomerase, with the **a**′ domain being a better oxidase [28].

Although Ero1p is essential for yeast (and *Caenorhabditis elegans*) viability, mice deficient in both Ero1α and Ero1β are viable [29], which stimulated the search for supplementary pathways of disulfide bond formation in the ER (reviewed in [30,31]). Alongside Ero1, additional sources of disulfide bond equivalents to PDI include peroxiredoxin IV [32,33], glutathione peroxidases [34] and vitamin K epoxide reductase [35]. The sulfhydryl oxidase QSOX can oxidize some substrates of the secretory pathway and extracellular matrix directly [36] and the selenoprotein Sep15 may also contribute to disulfide bond reduction/isomerization during glycoprotein quality control [37]. Low molecular weight thiols, principally glutathione, also regulate the redox balance of the ER [38,39].

## 3. The regulatory poise of Ero1α differs between cell types

**Figure 1.**
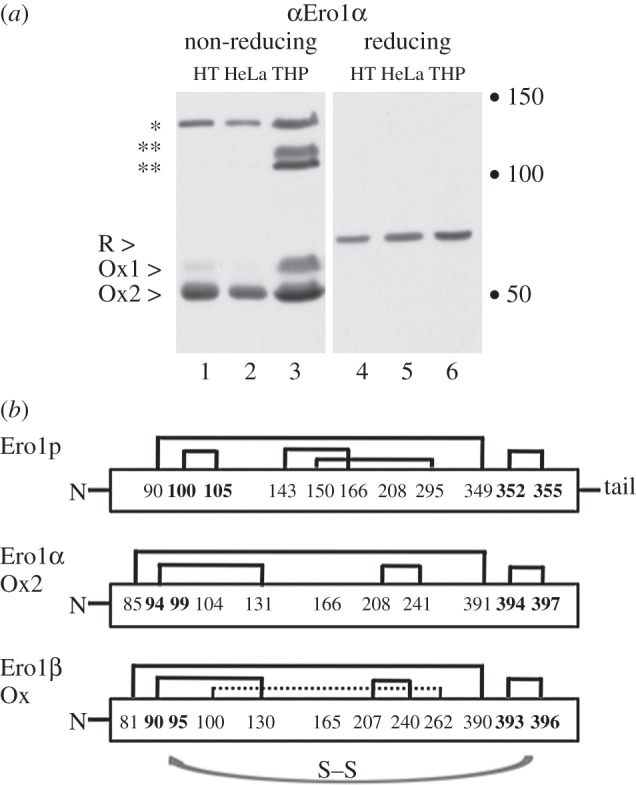
The redox state and interactions of Ero1α are cell-type dependent. (*a*) Equal amounts of lysates from HT1080 cells (lanes 1 and 4), HeLa cells (lanes 2 and 5) and THP1 cells (lanes 3 and 6) were analysed by non-reducing (lanes 1–3) or reducing (lanes 4–6) SDS-PAGE and probed for Ero1α expression. Endogenous disulfide-bonded complexes with PDI (asterisk) and other proteins (double asterisk) can be detected at steady state. Molecular weight markers of 50, 100 and 150 kDa are shown as dots. (b) A schematic of the disulfide bonds (black lines) of Ero1p, Ero1α (Ox2) and Ero1β (Ox). In Ero1α Ox2, the C94–C131 disulfide precludes formation of a disulfide at the active site C94–C99 (shown in bold). An analogous regulatory disulfide between C90 and C130 is found in Ero1β together with a likely additional long-range disulfide between C100 and C262 (hatched line). Note that the regulatory cysteines differ between Ero1α/β and Ero1p. The most N-terminal cysteine residues are not shown for simplicity. Disulfide bond flow from the C-terminal active site cysteines to the N-terminal active site cysteines (shown in bold) is depicted by a grey arrow.

Although the general flow of disulfides between Ero1 and PDI is now understood, many key points remain unknown. In higher eukaryotes, there are over 20 PDI homologues. Although some of them lack redox-active **a**-type domains, the majority are likely to be directly involved in disulfide bond formation or regulation [15,40]. In yeast, there is a hierarchy of interactions between Ero1p and the Pdi1p homologues [41], but it is not clear how many PDIs require Ero1 for the provision of disulfide bond equivalents in mammalian cells. Mammalian disulfide bond formation may also be regulated differently in diverse tissue types or physiological settings. To illustrate this, figure 1 shows an experiment in which the expression of Ero1α was analysed by Western blotting at steady state in three different cell lines, HT1080 (a fibrosarcoma), HeLa (a cervical carcinoma) and THP1 (a monocytic leukaemia). The proteins in the cell lysates were separated electrophoretically under non-reducing conditions (figure 1*a*, lanes 1–3), which allow disulfide-dependent interactions to be preserved, and reducing conditions, which instead disrupt disulfide bonds (figure 1*a*, lanes 4–6). It is known from previously published experiments that under non-reducing conditions, monomeric Ero1α can exist as a reduced form (R) and two partially oxidized forms, Ox1 and Ox2 [42]. The Ox2 form has a regulatory disulfide bond between C94 and C131 that inactivates the redox activity of Ero1α [23,43].

Endogenous Ero1α in HT1080 and HeLa cells was almost exclusively found in the compact Ox2 form (figure 1*a*, lanes 1 and 2) and represents an inactive reservoir of the protein. By contrast, THP1 cells expressed more of the active Ox1 form of Ero1α (figure 1*a*, lane 3). The fully reduced form of Ero1α was not detectable in either cell line at steady state by the 2G4 antibody. These cell lines do have a high secretory output, so why should they have a different Ero1α Ox1 : Ox2 balance? One possibility is that Ero1α is involved in a wider range of biological processes in monocytes and macrophages, which are professional antigen-presenting cells of the immune system. In support of this idea, it has been shown that Ero1α regulates the ER calcium channel IP3R and hence indirectly controls the release of calcium from the ER [44], a process that can induce apoptosis [45]. Ero1α localizes to mitochondrial-associated membranes (MAMs) under oxidizing conditions, where transfer of calcium can occur [44,46,47] and calcium sensing studies using fluorescent probes suggest that calcium levels respond to changes in Ero1α activity [48]. In cultured macrophages, Ero1α can subsequently induce the activation of the NADPH oxidase complex [49], which generates superoxide for the destruction of ingested pathogenic bacteria and mycobacteria. NADPH oxidase 2 function is important because genetic defects in components of the complex can lead to X-linked chronic granulomatous disease, which results in life-threatening bacterial infections [50]. It will be interesting to test whether NADPH oxidase activity can be controlled by modulating the Ero1α oxidation state, and to assess the relative contribution of Ero1α to calcium signalling compared with oxidative protein folding. Thus, it is becoming apparent that Ero1α function is not strictly limited to oxidative protein folding, but can contribute to multiple biochemical pathways, including cross-compartmental calcium fluxes and redox communication.

## 4. Ero1α engages in multiple disulfide-dependent interactions

Another possible explanation for cell-specific differences in Ero1α oxidation state is that Ero1α could be regulated by different PDI family members, such as ERp44, ERp57 and ERp72 [51–54], and these proteins may vary in their ability to reduce Ero1 regulatory disulfide bonds. In support of this idea, the experiment shown in figure 1*a* illustrates that Ero1α can be trapped in inter-molecular, disulfide-dependent complexes with different partners that vary depending on the cell type. Whereas Ero1α interacted equally well with a protein that is likely to be PDI (figure 1*a*, asterisk) in all three cell lines, additional inter-molecular Ero1α interactions can be seen in THP1 cells (figure 1*a*, lane 3, double asterisk). These interacting proteins have yet to be formally identified, but based on published and unpublished data, one is likely to be the PDI homologue ERp44, which was identified as a novel protein important for ER retention of Ero1α by the Sitia group [51]. ERp44 was subsequently shown to be involved in the quality control of the IgM immunoglobulin [55], adiponectin [56] and the serotonin receptor SERT [57]. These studies suggest that ERp44 is important for the assembly of proteins into oligomers, and probably acts as a platform upon which its clients are assembled prior to delivery to ER exit sites and post-ER compartments such as the ERGIC [58,59]. ERp44 is also important for the regulation of IP3R1 [60], which is of particular interest given the link between Ero1α and IP3R1 in macrophages discussed in the previous section. ERp44 mutants that bind Ero1α at high affinity inhibit oxygen consumption [25], but further work is required to determine how ERp44 directly regulates the oxidation state, and hence activity, of Ero1α in different cell types. However, experiments in transfected HeLa cells have shown that the interaction between ERp44 and Ero1α is independent of a hydrophobic hairpin in Ero1α that is required for full binding of Ero1α to PDI. PDI and ERp44, therefore, interact with Ero1α differently, providing scope for fine-tuning the activity of Ero1α in different cells and tissues [25].

## 5. Redox-sensitive regulation of Ero1α

**Figure 2.**
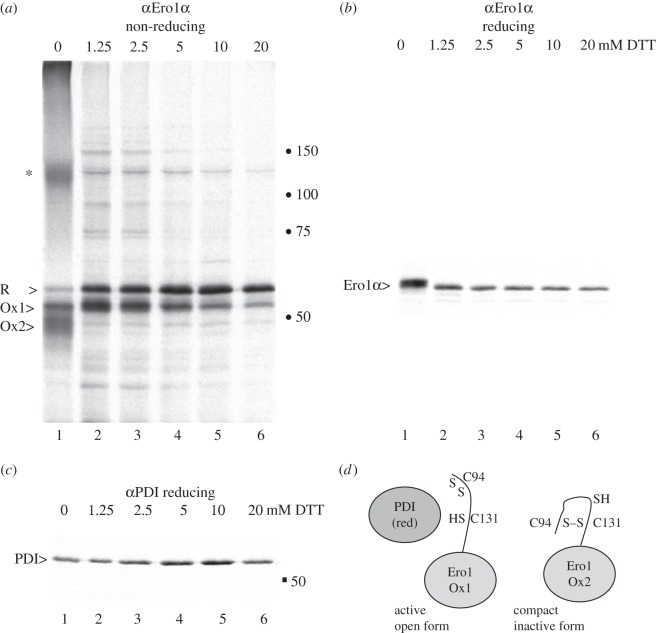
Selective reduction of Ero1α complexes. HeLa cells transfected with Ero1α were metabolically labelled for 5 min in the presence of 0, 1.25, 2.5, 5, 10 or 20 mM DTT and post-nuclear lysates subjected to immunoprecipitation using antibody D5. Samples were analysed on (*a*) non-reducing or (*b*) reducing 7.5% SDS-PAGE. The 120 kDa complex is indicated by an asterisk and molecular weight markers of 50, 75, 100 and 150 kDa are shown as dots. Alternatively, αPDI was used to retrieve PDI from the same lysates prior to analysis on reducing 7.5% SDS-PAGE (*c*). Ero1α R, Ox1 and Ox2 are indicated by greater than symbol (>). (*d*) Cartoon to illustrate the active ‘open’ form of Ero1α Ox1 compared with the more compact Ero1α Ox2. Ox1 lacks the regulatory C94–C131 disulfide and recruits reduced PDI for re-oxidation. For simplicity, only C94–C99 is shown. The higher apparent molecular weight of Ero1α in (*b*) lane 1 could be due to increased binding of NEM to more free cysteines, a change in the accessibility of a cysteine residue(s) to NEM or an alternative, redox-dependent post-translational modification at a cysteine residue.

**Figure 3.**
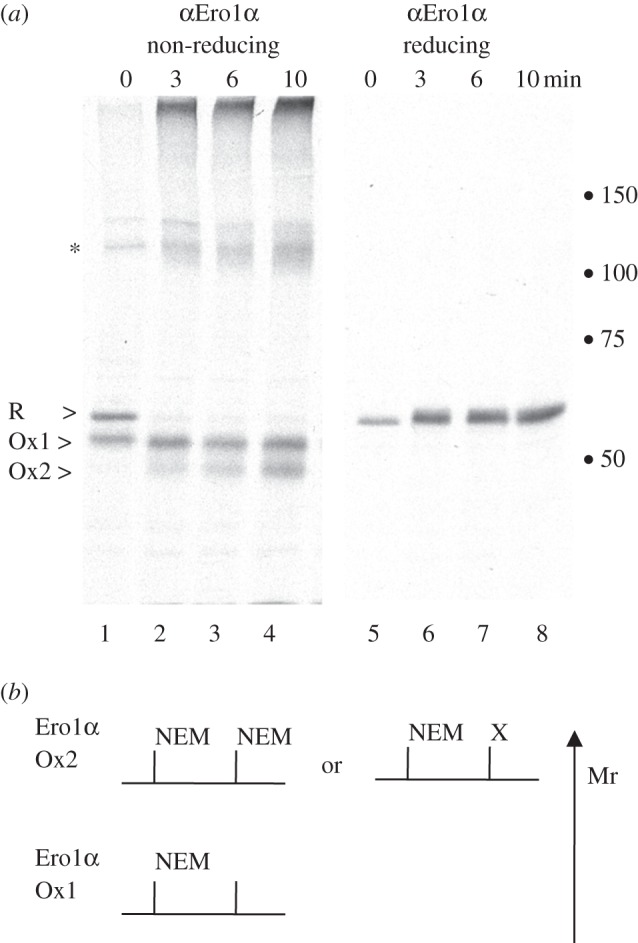
Post-translational oxidation of Ero1α. (*a*) HeLa cells transfected with pcDNA3.1-Ero1α were metabolically labelled for 5 min in the presence of 5 mM DTT and then chased for 0, 3, 6 or 10 min. Cell lysates were subjected to immunoprecipitation with D5 and analysed on non-reducing (lanes 1–4) or reducing (lanes 5–8) 7.5% SDS-PAGE. The Ero1α forms R, Ox1, Ox2 and the 120 kDa complex (asterisk) are shown. Dots represent 50, 75, 100 and 150 kDa markers. (*b*) Schematic to explain the mobility shift of Ero1α when analysed by reducing SDS-PAGE. After removal of DTT, Ox2 (and Ero1α-associated complexes) reappear and are more readily alkylated by NEM, increasing the molecular weight (Mr). X represents putative post-translational modifications that could also increase Ero1α molecular weight.

**Figure 4.**
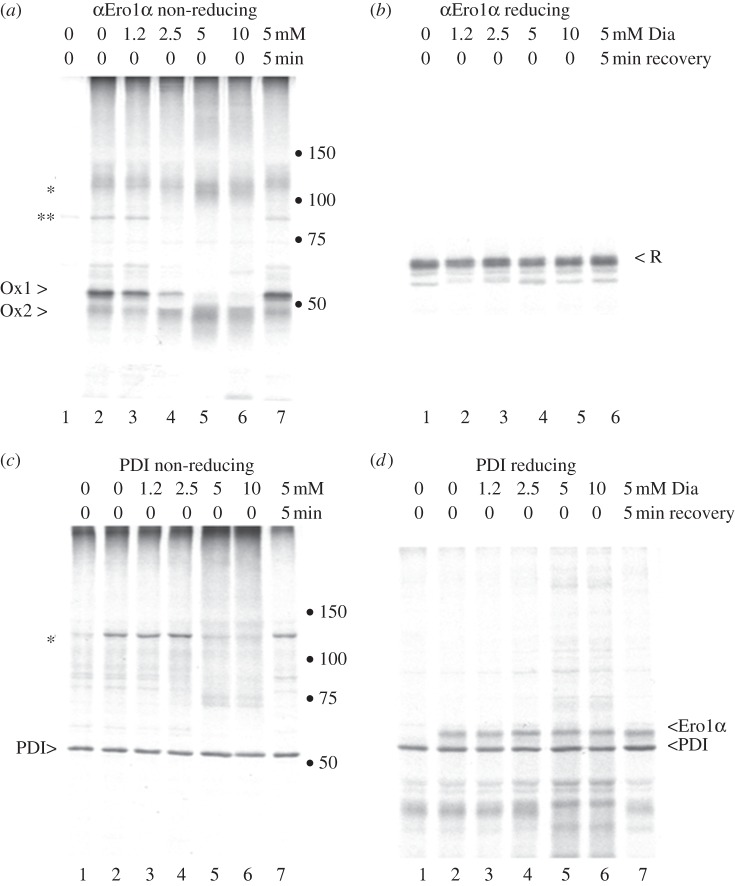
The response of Ero1α to an oxidative wave. HeLa cells transfected with pcDNA3.1-Ero1α (*a*,*c* and *d*, lanes 2–7; *b*, lanes 1–6) or mock-transfected cells (*a*,*c* and *d*, lane 1) were metabolically labelled in the presence of 5 mM DTT and then washed and chased for 10 min in the presence of 0, 1.2, 2.5, 5 and 10 mM diamide (Dia). A 5 mM diamide-treated dish then was allowed to recover by washing and chasing with normal chase medium for 5 min (*a*,*c* and *d*, lane 7; *b* lane 6). Ero1α (*a* and *b*) or PDI (*c* and *d*) were immunoprecipitated from the cell lysates and analysed on non-reducing (*a*,*c*) or reducing (*b*,*d*) 7.5% SDS-PAGE. Ero1α R, Ox1 and Ox2, together with the 120 kDa complex (asterisk) and an additional S–S linked complex (double asterisk) are indicated. Dots represent 50, 75, 100 and 150 kDa markers.

Elegant studies with yeast Ero1p [61–63], mammalian Ero1α [23,24,43,64] and mammalian Ero1β [65] have mapped out the regulatory disulfide bonds that control the activity of Ero1 proteins. These regulatory disulfides and their relationship to the redox-active cysteines are outlined in figure 1*b*. The C94–C131 regulatory disulfide bond in Ero1α ‘locks down’ residue C94; this prevents the active site C94–C99 disulfide from forming and subsequently donating a disulfide to PDI. The robustness of this intrinsic control system and its very rapid responsiveness to fluctuations in the redox environment is highlighted by three previously unpublished experiments from our laboratories (figures 2–4). HeLa cells transfected with Ero1α were exposed to various concentrations of the reducing agent dithiothreitol (DTT) in culture, radiolabelled and the DTT quenched with excess *N*-ethylmaleimide (NEM; figure 2). Analysis of the cell lysates by immunoprecipitation and non-reducing sodium dodecyl sulfate–polyacrylamide gel electrophoresis (SDS–PAGE) showed that the inactive oxidized Ox2 form of Ero1α was readily reduced when 1.25 mM DTT was added to the cell, whereas the partially oxidized (active) Ox1 form could resist reduction, with up to at least 20 mM DTT. Interestingly, a number of additional Ero1α disulfide-dependent complexes were revealed upon addition of 1.25 mM DTT (figure 2*a*, lane 2). These complexes may represent oligomers that reside in higher molecular-weight complexes during oxidizing conditions. The Ero1α associated proteins may be additional regulatory or accessory proteins (such as chaperones or PDI family members) that are recruited to Ox1 or to partially reduced Ero1α during the oxidation cycle. The bands in the 75–150 kDa region of the gel are likely to represent specific disulfide-dependent protein interactions with Ero1α because they are reduced by adding DTT to the sample buffer prior to SDS-PAGE (figure 2*b*).

As the DTT concentration applied to the living cell was increased, the intensity of the Ox2 signal declined (figure 2*a*, lanes 1–2), as did the total Ero1α signal (figure 2*b*, lanes 1–2). Because the signal of PDI (immunoprecipitated from the same lysates) remained similar between 0 and 1.25 mM DTT (figure 2*c*, lanes 1–2), the loss of signal was Ero1α specific. The loss of signal could reflect impaired detergent solubility in Triton X-100 owing to aggregation, loss of protein owing to degradation by ER quality control mechanisms or loss of antibody reactivity after post-translational modification. However, given the fact that the polyclonal antiserum recognizes a range of conformations and redox states of Ero1α, we favour the former explanation. Given the relationship between Ero1α and calcium signalling described above, it would be intriguing to determine whether the changes in Ero1α signal intensity relate to its recruitment to MAMs or to ER detergent-resistant membranes in a redox-dependent manner.

The DTT-induced change from Ox2 to Ox1 can be explained structurally by the transition of Ero1α from a compact form with an intact regulatory C94–C131 disulfide (Ox2) to a more ‘open’ active form (Ox1) in which the regulatory disulfide has been broken by DTT (figure 2*d*). The Ero1α C94–C99 disulfide is consequently primed to donate a disulfide to reduced PDI, which lacks regulatory disulfides.

## 6. Dithiothreitol-induced structural changes in Ero1α are reversible

To respond to changes in the redox environment, Ero1α must switch rapidly between active and inactive states. To assess how rapidly Ero1α altered its equilibrium when exposed to a redox shift, we investigated how the protein responded when oxidizing conditions were restored after a 5 min challenge with 5 mM DTT (figure 3). Ero1α partitions into R and active Ox1 during the 5 min pulse, with the Ero1α-PDI complex maintained under these conditions (figure 3*a*, lane 1). From previously published work, the Ero1α–PDI complex is likely to comprise both mature and radiolabelled PDI and Ero1α [42]. When DTT was washed out, the normal oxidation pattern of Ero1α was restored within 10 mins. The diffuse 120 kDa complex (*) and higher molecular weight complexes reformed within 3 min (figure 3*a*, lane 2). Re-oxidation of Ero1α resulted in rapid recovery of the inactive Ox2 form demonstrating that Ero1α is responsive to changes in the ER redox state.

Observation of the reducing gel showed an increase in the total Ero1α signal retrieved when cells were shifted from reducing to oxidizing conditions (figure 3*a*, lanes 5 and 6). Consistent with this experiment, the opposite pattern was seen when cells were shifted from oxidizing to reducing conditions (figure 2*b*, lanes 1 and 2). This observation shows that the decrease in signal in figure 2*b* cannot be explained by degradation of Ero1α. Ero1α became more accessible to NEM when the reducing agent was removed and the environment was made more oxidizing (compare figure 3*a*, lanes 5 and 6 with figure 2*b*, lanes 1–2), consistent with the finding that Ero1β FAD-binding site mutants make cysteines available in a temperature- or stress-dependent manner [66]. This somewhat counterintuitive finding is illustrated schematically in figure 3*b*. When Ero1α is covalently modified by NEM, it gains molecular weight and hence runs more slowly (higher up) in a reducing gel. Ero1α in the Ox2 form and/or Ero1α in higher molecular weight complexes must have either (i) more free cysteine residues than partially reduced Ero1α, (ii) cysteine residues that are less buried and hence more accessible to NEM, or (iii) cysteine residues that are subject to alternative post-translational modifications under oxidizing conditions, such as glutathionylation at an unpaired cysteine.

## 7. Ero1α interactions are differentially sensitive to oxidation cycles within the endoplasmic reticulum

The experiment in figure 3 shows how the Ero1α intramolecular redox switch can counterbalance changes in the ER redox state within minutes. Having shown that a reducing ER could alter the monomeric and oligomeric equilibrium of Ero1α, we investigated how Ero1α would respond when the ER was made more oxidizing. For this, we used the cell-permeable oxidant diamide, which can alter the redox potential of the cell by oxidizing glutathione [67]. Because diamide can decrease the amount of radiolabelling when added during the pulse, Ero1α-transfected HeLa cells were pulsed in the presence of DTT and then chased in the presence of diamide. These conditions allowed us to follow the fate of a synchronized Ero1α population and its response to an oxidative flux. Ero1α and PDI were immunoprecipitated from transfected HeLa cell lysates as before, prior to analysis on 7.5 per cent SDS-PAGE.

Figure 4 shows the result from such an experiment. Ero1α immunoprecipitations from mock-transfected cells were clear of signal (figure 4*a*, lane 1). After a pulse without a diamide chase, Ero1α existed as Ox1 and Ox2, a smeary 120 kDa form and some higher molecular weight complexes that likely include Ero1α-ERp44 (double asterisk; figure 4*a*, lane 2). When the chase was supplemented with increasing concentrations of diamide, Ox1 disappeared at the expense of the more compact Ox2 form (figure 4*a*, lanes 4–6). When Ero1α was allowed to recover from the diamide treatment by incubating the cells in normal chase medium, the protein returned to Ox1 (figure 4*a*, lane 7). The upper part of the non-reducing gels (figure 4*a,c*, lanes 2–7) showed that the diffuse 120 kDa complex persisted during diamide treatment, but also became more oxidized and compact. The complex returned to its original status when diamide was removed (figure 4*a*,*c*, lane 7). The reducing gel shows that in all lanes similar amounts of Ero1α were recovered when fully reduced in sample buffer (figure 4*b*, lanes 1–6). This result demonstrated that the various oxidized forms of Ero1α were not in a simple precursor–product relationship, but were in a dynamic equilibrium with each other that changed according to the redox status of the cell. Re-establishment of the *status quo* occurred within 5 min of removal of the oxidant.

When PDI was immunoprecipitated from the same cell lysates, it formed the expected approximately 120 kDa complex with Ero1α when visualized under non-reducing conditions (figure 4*c*, lane 2). Upon reduction, the Ero1α–PDI complex was disrupted and monomeric Ero1α was recovered (figure 4*d*, lane 2). However, when diamide was added at 5 mM or more, the dimeric 120 kDa PDI–Ero1α complex disappeared (figure 4*c*, lanes 5–6). Observation of the reducing gels (figure 4*d*, lanes 5 and 6) revealed that Ero1α could still be recovered in the PDI immunoprecipitates, indicating that PDI and Ero1α were interacting in the higher molecular weight disulfide-bonded complexes under these conditions. The 120 kDa PDI–Ero1α complex rapidly reappeared when diamide was washed out (figure 4*c*, lane 7).

The loss of the PDI–Ero1 dimer after 5 mM diamide treatment correlates with the loss of Ox1, consistent with the finding that Ox1 is the active form of Ero1α [43]. However, Ero1α complexes persisted after 5 and 10 mM diamide treatment (figure 4*a*, lanes 4–5, asterisk). This smear may include Ero1α homodimers or other as yet unidentified proteins that interact with and perhaps regulate Ero1α. The constant Ero1α signal in the αPDI immunoprecipitation (figure 4*d*) also suggests that PDI and Ero1α may be recruited to larger complexes when the ER becomes more oxidizing.

Strikingly, Ero1α–PDI dimers and the active Ox1 form of Ero1α are lost under oxidizing conditions when there is less need for de novo disulfide bond formation. The PDI–Ero1α pathway for disulfide bond formation is, therefore, sensitive to the redox state of the ER and may recruit different regulators during the redox cycle.

## 8. Implications for oxidative protein folding *in vivo*

Ero1α is an unusual example of a redox-active protein with conformation-dependent, DTT-resistant domains. Experiments from multiple laboratories, including ours, showed that Ero1 is redox regulated (reviewed in [68]). This is reflected in the relative distribution and dynamic response of Ero1α Ox1 (active) and Ox2 (inactive) to ER redox flux. PDI and Ero1α participate in a buffered feedback loop that maintains disulfide bond formation at an appropriate level when ER redox conditions fluctuate. Strongly reducing conditions disrupt Ero1β–PDI complexes at steady state [66,69], so it will be informative to directly compare the interactions of glycosylated Ero1α and Ero1β with PDI. *In vivo*, cells will not encounter DTT or diamide, but will be exposed to a range of physiological redox-active species; so how Ero1α and PDI respond to reactive oxygen species during hypoxia, nutrient flux and metabolic stress is an important question for the future.

Reduction of the Ero1 regulatory disulfides, by PDI or other mechanisms, is necessary for its activation [68]. Similarly, the redox-dependent Ox1–Ox2 transition from an active to an inactive form is likely to be important in preventing hyper-oxidation, which might be detrimental for reactions requiring PDI-dependent isomerization or reduction of substrates. Active Ox1 is decommissioned when required, supported by our experiments in which Ero1 rapidly and reversibly converts to Ox2 after diamide treatment (figure 4). The very strong inherent redox regulatory capacity of Ero1α has been confirmed by RNAi knockdown experiments in which the contributions of Ero1α, peroxiredoxin IV and vitamin K epoxide reductase (VKOR) to oxidative refolding of albumin were compared side by side [70]. Knockdown of Ero1α gave the most severe delay in recovery of oxidative protein folding, confirming that the Ero1–PDI pathway is the primary source of oxidizing equivalents. Whether this holds for all types of protein clients and all physiological conditions remains to be established.

## 9. Evidence for an oxidative protein folding ‘machine’ in the endoplasmic reticulum

Experiments presented here and in the literature show that Ero1α interacts specifically with PDI [42] and ERp44 [51] in disulfide-bonded complexes, and with itself as a homodimer [69]. Here, we show that other interactions are possible under mildly reducing conditions (figure 2). A number of discrete proteins disulfide-linked to Ero1α appear when the redox balance is altered. Whether these proteins are components of a larger ER-resident machine for the control of oxidative protein folding is open to question. One possibility is that other proteins involved in disulfide bond formation and regulation such as peroxiredoxin IV, glutathione peroxidases and VKOR are brought together with PDI and Ero1α, perhaps to ER subdomains in a redox-dependent manner. In support of this idea, we note from many of our Ero1α immunoprecipitation and blotting experiments that complexes resolve towards the top of the stacking gel on non-reducing SDS-PAGE; these complex(es) readily reform when normal conditions are restored after redox flux (e.g. figure 3). Although it is possible that these complexes contain misfolded Ero1α, the expression levels of Ero1α in transfection experiments are comparable with endogenous levels of Eros in some tissues [29,69] and after induction of Ero1α by the unfolded protein response or by hypoxia [71,72]. Disulfide trapping combined with SDS-PAGE is an excellent tool for identifying potential redox-active partnerships, but it cannot discriminate between different higher-order complexes that may exist under native conditions. Some attempt has been made to probe the nature of Ero1 complexes *in vivo* using gel filtration. For example, analysis of Ero1β from the stomach and pancreas, where Ero1β is highly expressed, shows that the majority of Ero1β elutes with a profile consistent with that of a complex [69].

Other chaperone networks in the ER have been detected, with BiP (Grp78) a key protein hub for mediating interactions with components of the translocation, protein folding and stress sensing machineries (e.g. [73–75]). By associating with different PDI family members, BiP can be involved in both productive oxidative protein folding (by associating with PDI) and in reductive unfolding for protein degradation (by associating with ERdJ5) [76,77]. BiP may be able to multi-task partly because of regulation by post-translational modifications: ADP-ribosylation of BiP has recently been shown to be important for BiP involvement in the unfolded protein response [78]. However, our understanding of the interplay between different ER chaperones remains incomplete. As an example, Jansen *et al.* [79] have proposed an interaction map for ER chaperones that highlights a hitherto unappreciated role of cyclophilins in the function of PDI proteins. Cyclophilin B can interact with at least PDI, ERp72 and P5 and there are additional interactions between ER-localized FK-binding proteins and ERp57, ERp29 and ERp19. It is clear that different protein folding complexes exist in the ER and it will be interesting to see how Ero1 proteins functionally relate to these networks, particularly during times of physiological stress or high secretory demand.

## 10. Material and methods

### (a) Cell lines

The monocytic cell line THP1 (gift from J. Robinson) was maintained in Roswell Park Memorial Institute medium, the fibrosarcoma HT1080 was maintained in Dulbecco's modified Eagle's medium (DMEM) and the human cervical carcinoma cell line HeLa was maintained in DMEM with non-essential amino acids. The cell lines were supplemented with 8 per cent fetal calf serum (FCS), 100 units ml^−1^ penicillin, 100 μg ml^−1^ streptomycin and 2 mM glutamax and maintained at 37°C and 5 per cent CO_2_.

### (b) Antibodies and cDNA

The polyclonal anti-PDI serum has been described previously [42]. The polyclonal antiserum D5 was raised against non-reduced, reduced and denatured forms of an amylose resin-purified, mannose binding protein–Ero1α fusion protein (New England Biolabs) expressed in *Escherichia coli* [79]. The monoclonal antibody 2G4 was raised against recombinant full-length Ero1α [59]. The construction and sequencing of the Ero1α cDNA behind the T7 promoter in pcDNA3.1 has been previously described [9].

### (c) Transfections

HeLa cells cultured in 6 cm dishes were transiently transfected with 2 μg pcDNA3.1-Ero1α mixed with 10 μl lipofectin (Invitrogen) according to the manufacturer's instructions.

### (d) Detection of endogenous Ero1α

HeLa, HT1080 and THP1 cells were lysed in 600 μl of lysis buffer (20 mM MES, 30 mM Tris, 100 mM NaCl, pH 7.4), with 1 per cent Triton X-100, 10 µg ml each of chymostatin, leupeptin, antipain and pepstatin supplemented with 20 mM NEM as an alkylating agent. Post-nuclear supernatants were prepared by centrifugation at 16 100*g* for 10 min at 4°C and equal amounts of protein (Bradford assay) were loaded onto SDS-PAGE in Laemmli sample buffer with or without 50 mM DTT as a reducing agent. Proteins were transferred to polyvinylidene difluoride membranes for 2 h and immunodetection was performed using 2G4 Mab tissue culture supernatant as the primary antibody, and 1 : 3000 GAMPO (Dako) as the secondary antibody. Proteins were visualized by enhanced chemiluminescence (GE Healthcare) and exposure to film (Kodak).

### (e) Metabolic labelling and pulse-chase analysis

Sub-confluent HeLa cells in 6 cm dishes were starved with MEM lacking cysteine and methionine (Invitrogen) for 30 min, pulse-labelled for the times stated with 10 μCi [^35^S]-labelling mix per dish and subsequently chased when necessary with complete medium supplemented with 5 per cent FCS, 10 mM HEPES pH 7.4, 5 mM methionine, 5 mM cysteine and 1 mM cycloheximide. At given time intervals, the chase was stopped by flooding the cells with ice-cold HBSS (Invitrogen) supplemented with 20 mM NEM to trap folding intermediates. In some experiments, freshly prepared DTT or diamide (Sigma) solutions were added to the pulse or chase medium, as stated. The cells were lysed in 600 μl of lysis buffer (20 mM MES, 30 mM Tris, 100 mM NaCl, pH 7.4), containing 1 per cent Triton X-100, 10 µg ml^−1^ each of chymostatin, leupeptin, antipain and pepstatin A, 1 mM PMSF, and 20 mM NEM and the nuclei removed by centrifugation for 10 min at 4°C and 16 000 *g*. Immunoprecipitations were performed at 4°C for either 2 h or overnight using antibodies immobilized on 30 μl of a 10 per cent suspension of protein A sepharose beads. Collected complexes were washed twice at room temperature in wash buffer (300 mM NaCl, 0.05% Triton X-100 and 0.05% SDS, 10 mM Tris-HCl, pH 8.6) prior to uptake in sample buffer. After a 3 min incubation at 95°C, half the samples were reduced with 50 mM DTT, and the proteins were analysed by 7.5 per cent SDS-PAGE.
